# Supplementary material for: The microtubule targeting agent ST-401 triggers cell death in interphase and prevents the formation of polyploid giant cancer cells
Source: J Transl Med. 2024 May 10;22:441. doi: 10.1186/s12967-024-05234-3 (PMC11084142; doi:10.1186/s12967-024-05234-3)
Supplement: Supplementary file 1 — Additional file 1: Additional figures. [file 12967_2024_5234_MOESM1_ESM.pdf]

Mitosis exit followed by death in interphase prevents the development of polyploid giant cancer cells:

Juan Jesus Vicente, Kainat Khan, Grant Tillinghast, José L. McFaline-Figueroa,
Yasemin Sancak and Nephi Stella

Supplementary Figures

Figure S1a-b: GI50, TGI and LC50 MeanGraph and concentration/response curves for ST-401.

Figure S1c: NCI60 Mean Graphs – 12 highest correlations between ST-401 and Approved Agents (169 NSC)

Figure S1d: Tumorigenesis parameters of cancer cell line in the NCI-60 expressed as a function of ST-401 induced TGI.

Figure S3a: Four h treatment with NOC disrupts the cell cycle of HCT-116: milder response by ST-401.

Figure S3b-d: NOC treated cells are bigger than ST-401 treated cells after 24h.

Figure S4a-b: Representative images of cells dying under drug treatment, and quantification of cell death before and after mitosis.

Figure S4c-f: Quantification of apoptosis/necrosis levels in HCT116 treated cells measured by flow cytometry.

Figure S4g: NOC trigger apoptosis: milder response by ST-401.

Figure S4h-j: NOC treated cells with increased autophagosomes are bigger than ST-401 treated cells after 24h.

Figure S5a-d: Overview of single-cell RNA-seq sample metrics.

Figure S5e-f: Summary of differential gene expression analysis of vehicle, NOC and ST-401 exposed cells.

Figure S5g-h: Gene-enrichment analysis at 24h using Metascape.

Figure S5i: Dimensionality reduction using Principal Component Analysis (PCA) analysis of the cluster distribution of CTR, NOC and ST-401 treated HCT116 cells for 8h and 24h.

Figure S6a-b: Ponceau Red staining of gels shown in Figure 6.

Figure S7a-b: NOC (100 nM) and ST-401 (100 nM) treatment for 24h affects spare capacity and proton leak in HCT-116 cells.

Figure S8a-b: NOC (100 nM) and ST-401 (100 nM) treatment for 24h affects non-mitochondrial respiration and maximal respiration as measured by Cell Mito Stress Test, in SF-539 and SNB-19 cells.

Figure S9a-b: NOC (100 nM) and ST-401 (100 nM) treatment for 24h affects mRNA encoding for immune responses in HCT-116 cells.

Supplementary Figures S1a-b: GI50, TGI and LC50 MeanGraph and concentration/response curves for ST-401: **a)** MeanGraph representation of the concentrations in the NCI60 cell lines for ST-401 to cause GI50 (50% growth inhibition), TGI (total growth inhibition) or LC50 (50% cell kill) in the screen. The midline for each graph is the average concentration for all of the cell lines at that endpoint. Thus, sensitivity cell lines are identified by bars drawn to the right of the average concentration for all of the cell lines at that endpoint. **b)** The concentration/response data are also presented. Correlations among the three endpoint patterns were low. The TGI endpoint was used for subsequent work since the TGI endpoint has been found to be a better indicator for mechanisms which target tubulin, and a lower correlation limit of 0.6 was used for significance.

a

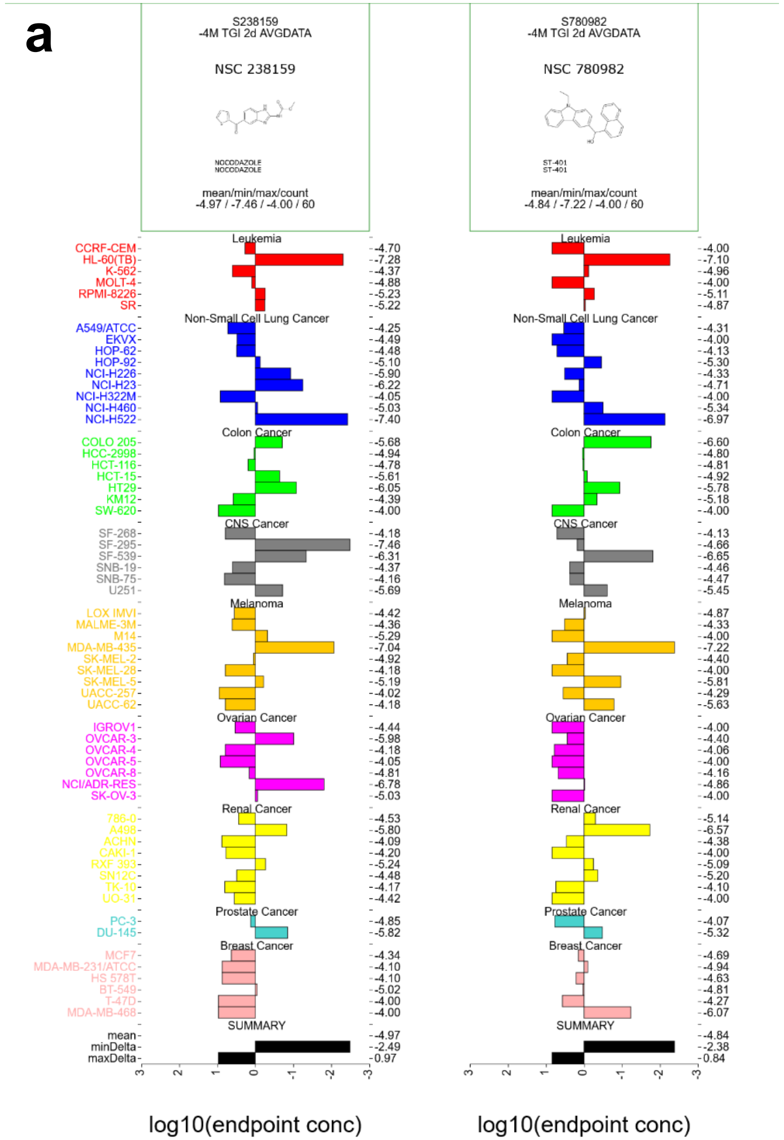

b

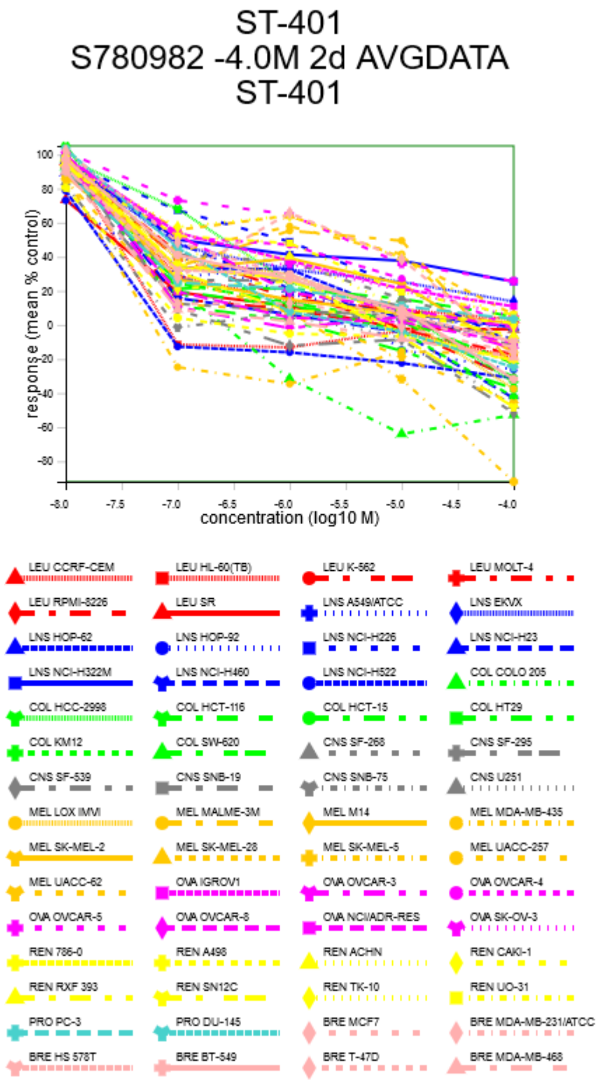

Supplementary Figure S1c: NCI60 Mean Graphs – 12 highest correlations between ST-401 and Approved Agents (169 NSC): MeanGraph showing the TGI patterns for ST-401 and the most-highly correlated TGI patterns in the NCI60 set of Approved Agents, ordered by decreasing correlation.

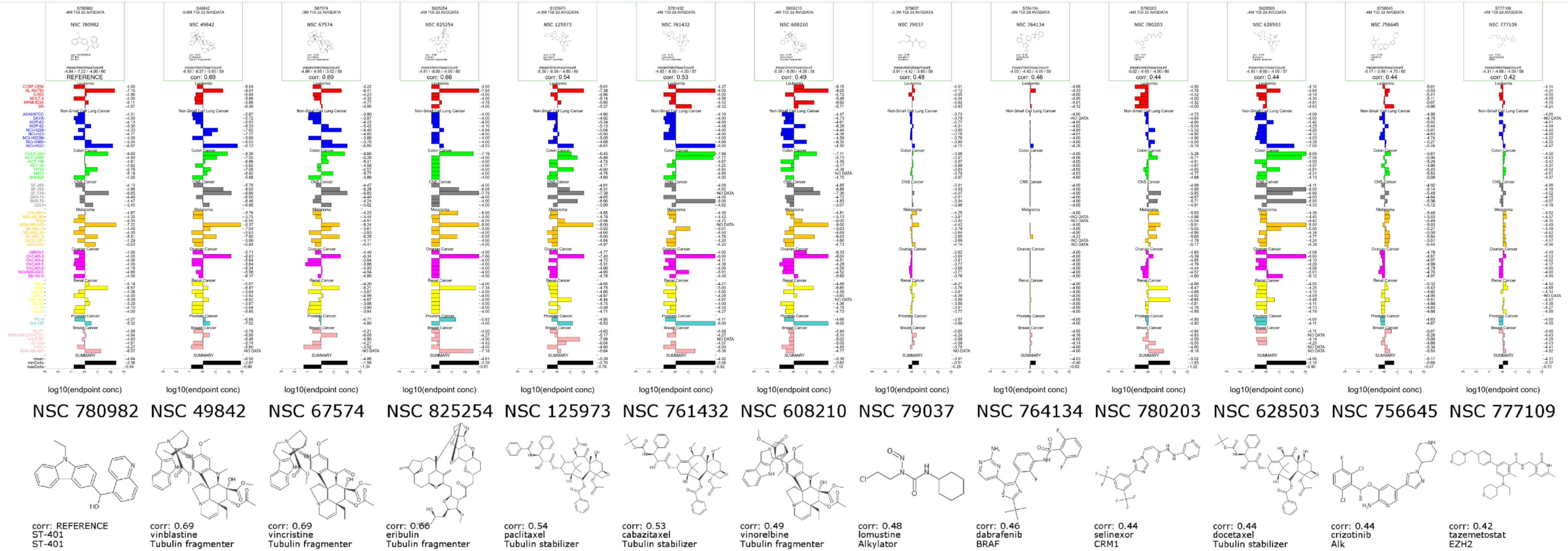

Supplementary Figure S1d: Tumorigenesis parameters of cancer cell line in the NCI-60 expressed as a function of ST-401 induced TGI: Table provides the sensitivity expressed at TGI of the first 31 cell lines that are most sensitive to ST-401 (from MDA-MB-435 to SNB-19 cells). Also shown is whether the cell lines are wild type or p53 mutants, their modal number of chromosomes (from 43 to 116) and their doubling proliferation time (from 17h to 80h).

Cancer Type	Cell Line	ST-401	TP53	M	Doubling
		(TGI)	(WT/Mut)	(#)	(h)
Melanoma	MDA-MB-435	5.8E-08	WT	56	26
Leukemia	HL-60(TB)	7.9E-08	Mut	45	29
Renal	A498	1.4E-07	WT	74	67
Colon	COLO 205	2.1E-07	Mut	72	24
NSCL	NCI-H522	2.2E-07	Mut	51	38
CNS	SF-539	3.1E-07	Mut	88	35
Melanoma	SK-MEL-28	1.5E-06	Mut	100	25
Melanoma	SK-MEL-5	1.5E-06	WT	88	35
Prostate	DU-145	2.1E-06	Mut	59	32
Colon	HT29	2.3E-06	Mut	67	20
Melanoma	UACC-62	2.3E-06	WT	73	31
NSCL	HOP-92	4.5E-06	Mut	94	80
Leukemia	RPMI-8226	5.7E-06	Mut	64	34
Renal	786-0	6.7E-06	Mut	83	22
Breast	MDA-MB-468	8.5E-06	Mut	64	62
Colon	KM12	1.0E-05	Mut	43	24
Colon	HCT-15	1.1E-05	Mut	44	21
Colon	HCT116	1.1E-05	WT	45	17
NSCL	NCI-H460	1.1E-05	WT	53	18
Ovarian	NCI/ADR-RES	1.2E-05	WT	65	34
Melanoma	LOX IMVI	1.2E-05	WT	64	21
Leukemia	SR	1.3E-05	WT	46	29
Breast	MCF7	1.4E-05	WT	65	25
CNS	SF-295	1.5E-05	Mut	116	30
CNS	U251	1.5E-05	Mut	52	24
Renal	SN12C	1.7E-05	Mut	64	30
Breast	HS 578T	1.8E-05	Mut	57	54
Colon	HCC-2998	2.0E-05	Mut	44	32
Breast	MDA-MB-231	2.3E-05	Mut	54	42
Renal	ACHN	2.5E-05	WT	51	28
CNS	SNB-19	3.4E-05	WT	61	35

Supplementary Figure S3a: Four h treatment with NOC disrupts the cell cycle of HCT-116: milder response by ST-401. Data were analyzed by ANOVA multi-comparison test followed by DUNETT's post test. N = 3 independent experiments. * = P < 0.0332, ** = P < 0.021, *** = P < 0.0002 and **** = P < 0.0001 different from Vehicle.

a

4 h	Sub-G1			G1			S			G2/M			Over-G2		
	Mean	SD	p	Mean	SD	p	Mean	SD	p	Mean	SD	p	Mean	SD	p
**CTR**	0.9	0.2		51.2	3.9		21.1	2.1		23.7	1.8		3.1	1.4	
10nM ST	1.3	1.0	ns	49.2	4.9	ns	22.2	2.6	ns	23.2	2.7	ns	4.0	1.4	ns
30nM ST	0.9	0.2	ns	47.5	4.6	ns	22.9	2.1	ns	24.3	2.9	ns	4.5	1.1	ns
50nM ST	0.9	0.4	ns	47.2	3.8	ns	23.0	2.2	ns	24.5	2.7	ns	4.4	0.8	ns
100nM ST	2.3	0.8	**	31.2	1.6	****	26.7	2.1	*	35.8	3.9	**	3.9	0.9	ns
300nM ST	1.4	0.3	ns	20.6	1.3	****	29.5	4.4	**	44.8	5.1	**	3.8	1.9	ns
10nM NOC	1.2	0.2	ns	45.7	5.2	ns	22.1	2.1	ns	26.0	4.1	ns	5.0	0.8	ns
30nM NOC	1.9	1.0	*	26.8	2.6	**	27.3	2.4	*	37.5	6.5	**	6.5	3.1	ns
50nM NOC	1.6	0.9	ns	20.1	2.7	**	29.0	4.5	**	41.8	6.8	**	7.5	2.8	**
100nM NOC	0.8	0.2	ns	13.8	3.4	**	31.1	2.9	**	47.4	1.3	**	6.9	2.4	*
300nM NOC	0.7	0.1	ns	14.5	2.7	**	30.6	2.2	**	46.6	1.1	**	7.7	2.7	**

Supplementary Figure S3b-d: NOC treated cells are bigger than ST-401 treated cells after 24h. **b)** Cell treated with CTR, NOC or ST (100 nM) were analyzed by flow cytometry using the classical FSC versus SSC signal to discriminate cell size. The percentage of cells in Region B was measured and considered as cells "bigger than normal" (Region A). **c)** Quantification of Region B. Bars are median with 95% CI. **d)** Distribution of cell size (FSC-A in arbitrary units). N = 3 independent experiments.

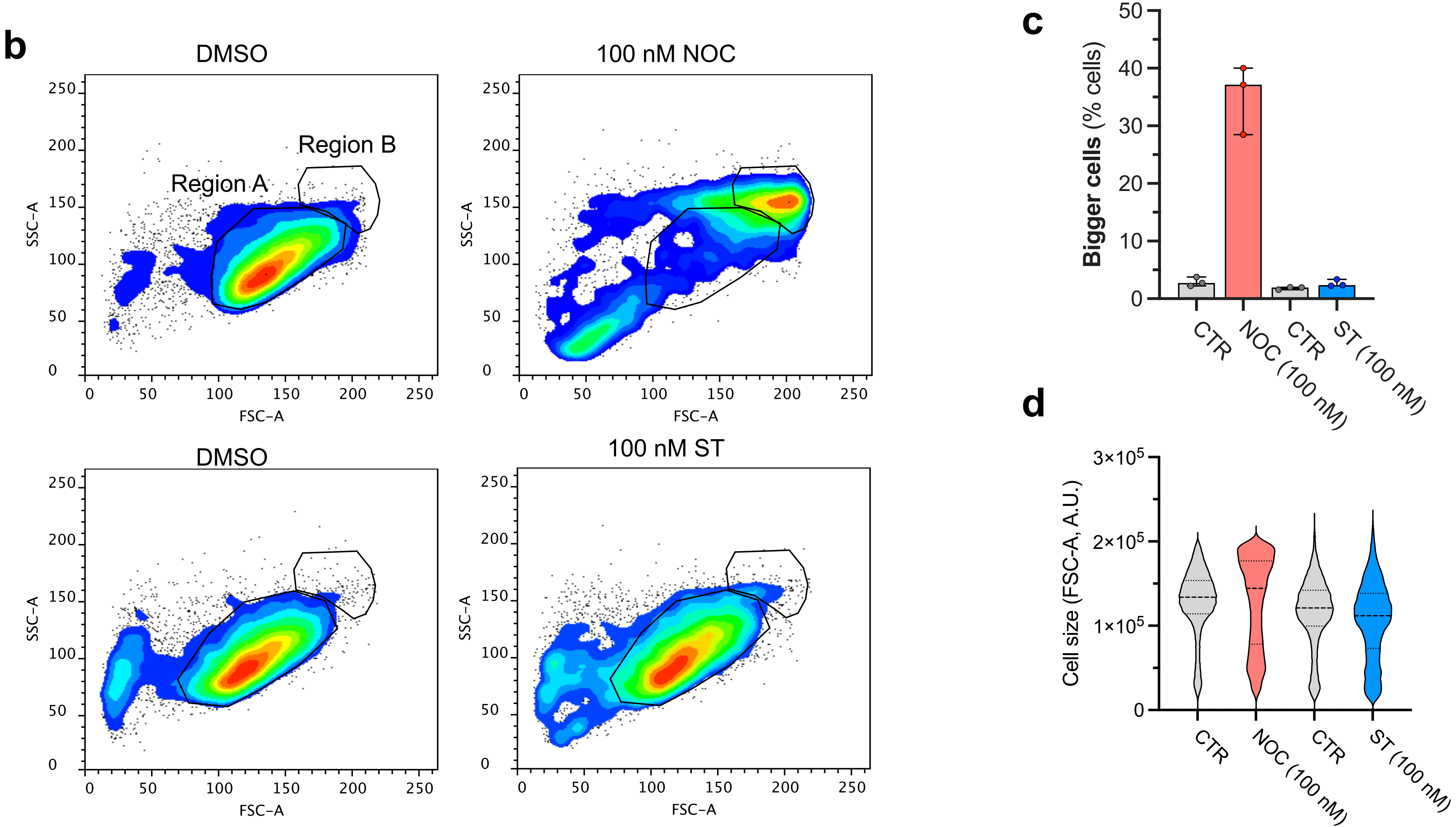

Supplementary Figure S4a-b: Representative images of HCT116 cells dying in response to ST-401 and NOC treatment, and quantification of death before and after mitosis during 24h treatment analyzed by live cell time-lapse microscopy. **(a)** HCT116 cells expressing H2B-mCherry were recorded for 24h following treatment with either ST-401 (100 nM) or NOC (100 nM). The cell in the top row illustrates ST-401 induced death in interphase. The cell in the bottom row illustrates NOC induced death in mitosis. The time t = 0 for cell in mitosis was set when nuclear envelope breaks down. **(b)** Live cell time-lapse movies were visually analyzed and cell death before, during and after mitosis manually quantified. Data are expressed as percentage of total cell death from 3 independent experiments (n = 589 cells for ST-401 and 1228 cells for NOC).

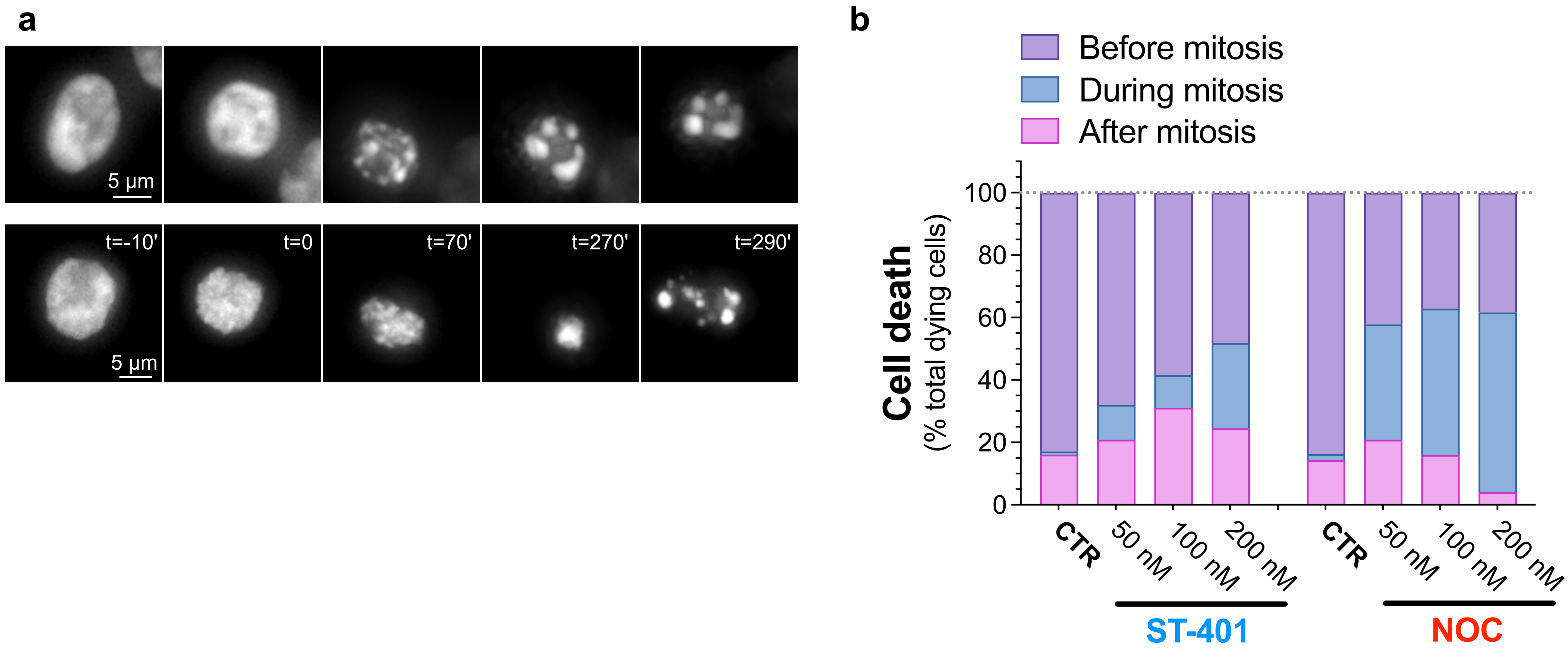

Supplementary Figures S4c-f: Quantification of apoptosis/necrosis levels in HCT116 treated cells measured by flow cytometry. **b-d)** Quantification of % cells in necrosis and/or apoptosis from flow cytometry data. **e)** Representative graphs of apoptosis/necrosis experiments at 100 nM. At 3 and 5 days we can see an increase in the number of cells at high FITC-A fluorescence, corresponding with high levels of Annexin V of cells going through apoptosis. Cells undergoing necrosis label A- and PI+, while labeling for A+ and PI- indicates early-apoptosis and A+/PI+ indicates late-apoptosis.

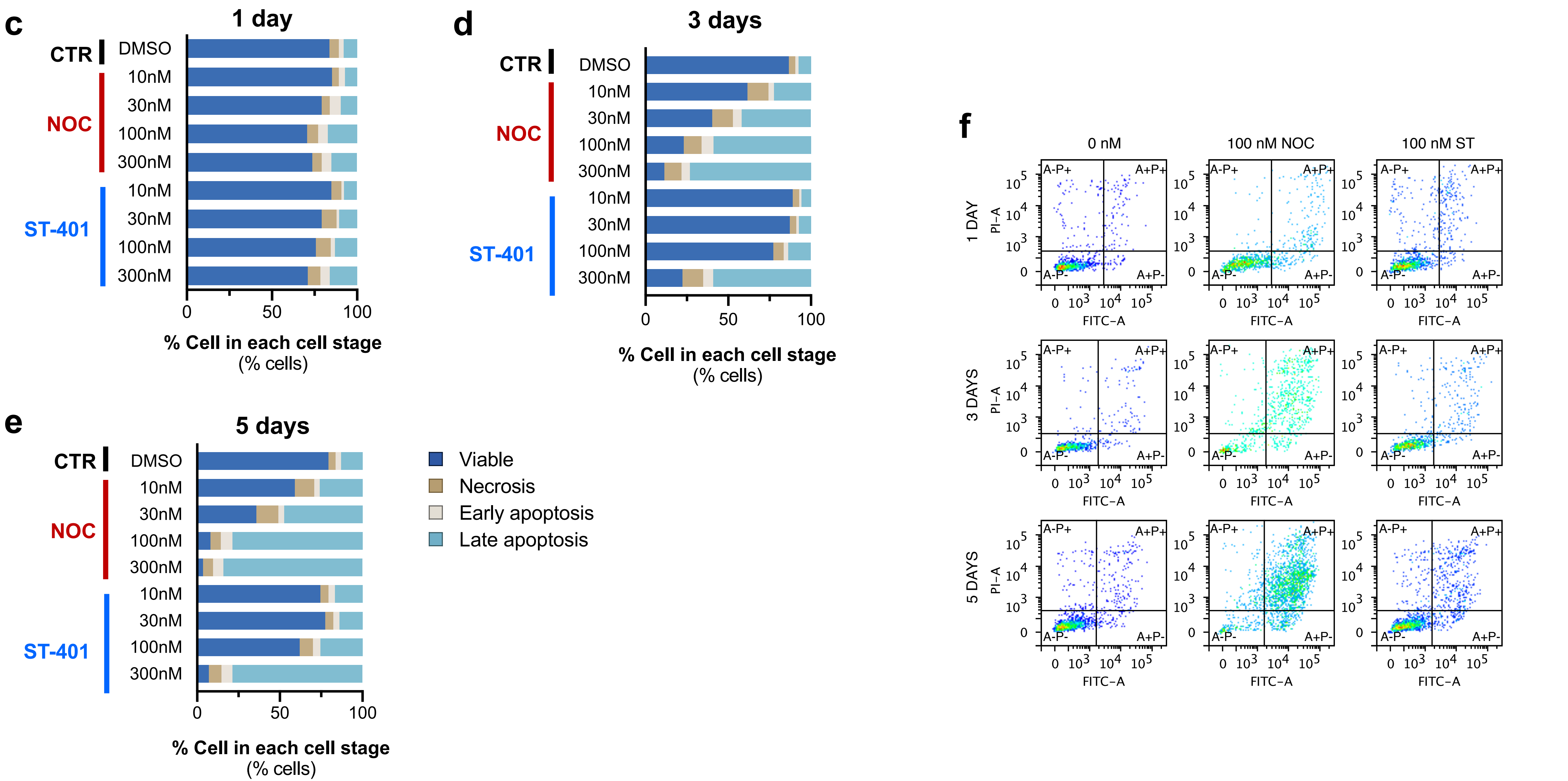

Supplementary Figure S4g: NOC trigger apoptosis: milder response by ST-401**.** Data were analyzed by ANOVA multi-comparison test followed by DUNETT's post test. $N = 3$ independent experiments. * = $P < 0.0332$, ** = $P < 0.021$, *** = $P < 0.0002$ and **** = $P < 0.0001$ different from Vehicle (CTR).

1 day	Viable			Necrosis			Early apoptosis			Late apoptosis		
	Mean	SD	p	Mean	SD	p	Mean	SD	p	Mean	SD	p
DMSO	83.62833	3.645125		5.695	2.095898		2.706667	1.533749		7.973333	2.163235	
10nM ST	84.92333	1.930423	ns	5.84	2.181582	ns	1.42	0.130767	ns	7.813333	0.550485	ns
30nM ST	79.01	0.913947	ns	8.833333	2.306281	ns	1.533333	0.15308	ns	10.62	1.537173	ns
100nM ST	75.78333	5.229812	ns	8.61	2.372109	ns	2.596667	0.67159	ns	13.01333	2.820591	ns
300nM ST	71.00333	1.831784	**	7.413333	1.59707	ns	5.496667	0.99143	*	16.08333	0.725695	**
10nM NOC	85.24667	4.737767	ns	3.943333	0.882968	ns	3.733333	1.916907	ns	7.076667	2.17238	ns
30nM NOC	79.15667	4.259018	ns	4.833333	1.158462	ns	6.29	2.362478	**	9.723333	1.348122	ns
100nM NOC	70.57667	10.19993	**	6.533333	1.767946	ns	5.693333	1.070389	*	17.19333	7.700424	**
300nM NOC	73.69667	1.260569	*	5.333333	1.568991	ns	5.903333	1.024809	*	15.06333	0.82282	*

3 days	Viable			Necrosis			Early apoptosis			Late apoptosis		
	Mean	SD	p	Mean	SD	p	Mean	SD	p	Mean	SD	p
DMSO	86.61	2.59513		3.786667	1.155436		1.996667	0.325167		7.606667	1.669441	
10nM ST	88.92667	3.2171	ns	3.886667	2.066454	ns	1.47	0.610492	ns	5.72	1.795188	ns
30nM ST	87.06	2.781924	ns	3.97	1.51	ns	1.646667	0.754476	ns	7.33	2.536553	ns
100nM ST	77.18333	6.473054	ns	6.226667	3.242628	ns	2.733333	0.785642	ns	13.85333	3.995552	ns
300nM ST	22.33667	2.061173	****	12.49	3.461344	*	6.003333	2.115569	ns	59.17667	4.139328	****
10nM NOC	61.51	9.880911	**	12.62667	0.453468	*	3.43	2.137124	ns	22.43667	8.147443	*
30nM NOC	40.32667	14.54225	****	12.42667	5.645754	*	5.16	2.06182	ns	42.08	9.078128	****
100nM NOC	23.05333	4.275714	****	10.73667	4.134941	ns	7.323333	2.854021	*	58.89667	5.965252	****
300nM NOC	11.26333	5.153565	****	10.36333	3.14945	ns	5.053333	2.371863	ns	73.32333	5.237694	****

5 days	Viable			Necrosis			Early apoptosis			Late apoptosis		
	Mean	SD	p	Mean	SD	p	Mean	SD	p	Mean	SD	p
DMSO	79.28333	1.696654		4.403333	1.475545		3.406667	1.301166		12.90667	1.92962	
10nM ST	74.54667	4.735149	ns	4.756667	1.030162	ns	4.013333	1.54442	ns	16.68667	2.804039	ns
30nM ST	77.32667	2.916099	ns	4.996667	1.310432	ns	3.703333	1.720533	ns	13.97667	2.621475	ns
100nM ST	61.97	9.258164	ns	8.093333	2.590103	ns	4.39	2.953032	ns	25.55	8.851107	ns
300nM ST	7.09	0.818352	****	7.613333	3.012812	ns	6.52	0.238118	ns	78.77333	2.906636	****
10nM NOC	59.07667	24.96963	ns	11.67667	8.382722	ns	3.353333	0.942992	ns	25.89333	16.83052	ns
30nM NOC	35.80333	17.35193	***	13.21333	4.825022	ns	3.5	0.915369	ns	47.48333	19.6458	**
100nM NOC	7.956667	0.647482	****	6.406667	2.185002	ns	6.933333	1.254804	ns	78.70333	0.776938	****
300nM NOC	3.38	0.301993	****	6.14	2.865118	ns	6.29	0.746458	ns	84.18667	2.917693	****

Supplementary Figure S4h-j: NOC treated cells with increased autophagosomes are bigger than ST-401 treated cells after 24h. **g-i)** Cells treated with CTR, NOC or ST (100 nM) were analyzed by flow cytometry using the classical FSC versus SSC signal to discriminate cell size, and for fluorescence intensity of MDC (for autophagy). The cells in the region M1 (cells positive for autophagosomes) appear at higher FSC (size) and SSC (internal complexity/granularity) values in top panels (red spots). Representative result of one out of three independent experiments.

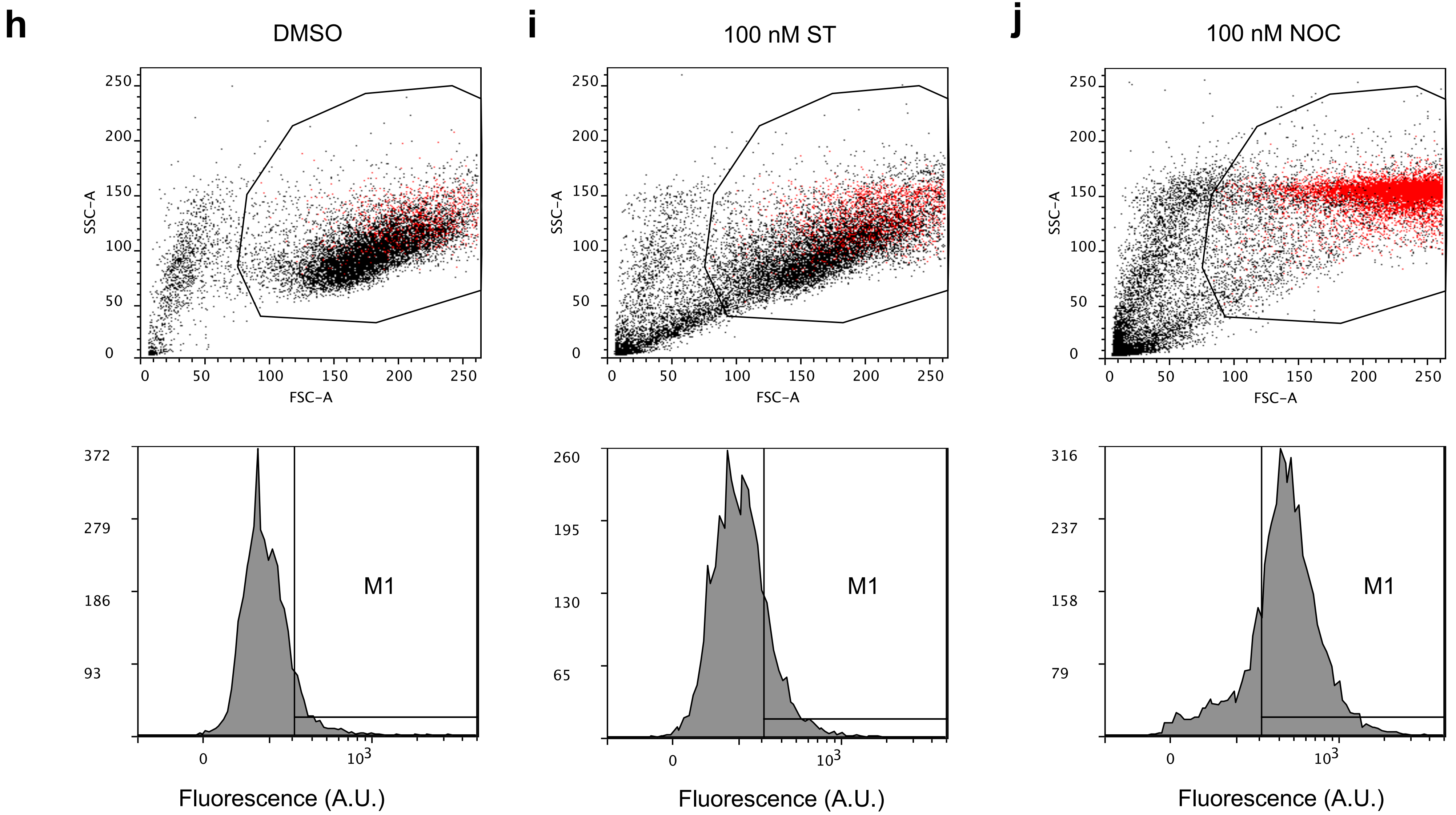

Figure S5a-d: Overview of single-cell RNA-seq sample metrics. **a)** Boxplots of the number of unique molecular identifiers (UMIs) for the cells in our experiment. **b)** Violin plots of the percentage of mitochondrial reads per cell) for the cells in our experiment. **c)** Summary of the total cell number per sample. **d)** Correlation or replicate expression values (Pearson's rho).

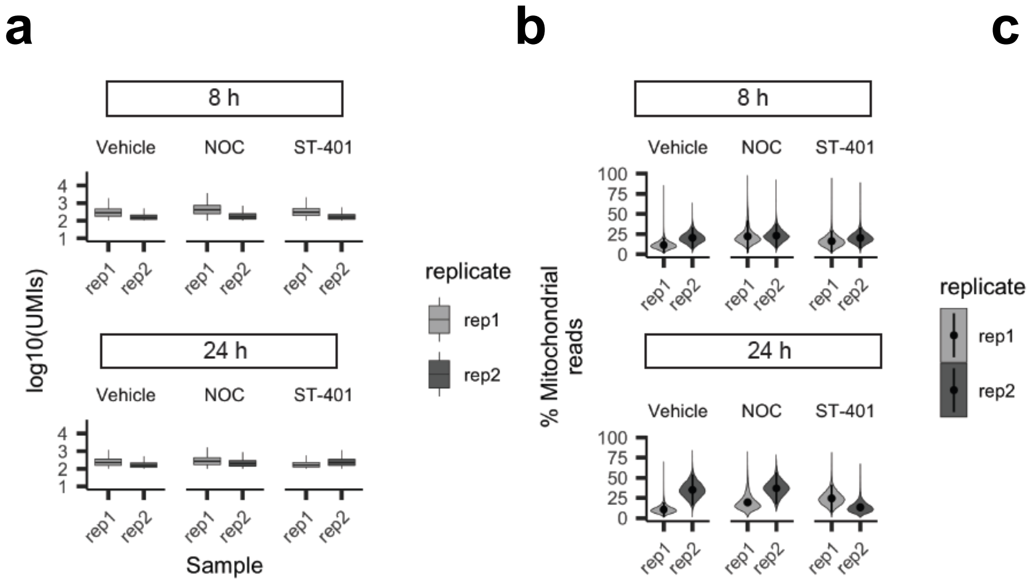

c

Final cell counts (> 100 UMIs, < 25% mitochondrial reads)			
Treatment	Time point	Replicate	Total cells
Vehicle	24	Rep 1	43926
Vehicle	24	Rep 2	2327
Vehicle	8	Rep 1	29022
Vehicle	8	Rep 2	6518
NOC	24	Rep 1	33027
NOC	24	Rep 2	219
NOC	8	Rep 1	19124
NOC	8	Rep 2	2768
ST-401	24	Rep 1	13431
ST-401	24	Rep 2	26854
ST-401	8	Rep 1	28907
ST-401	8	Rep 2	8042

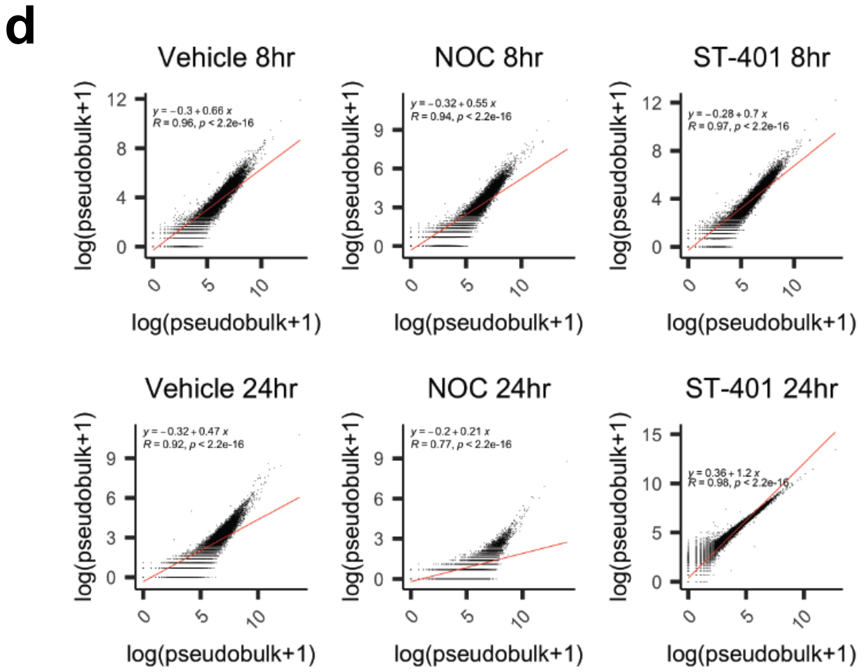

Supplementary Figures S5e-f: Summary of differential gene expression analysis of vehicle, NOC and ST-401 exposed cells. **e)** Volcano plots of the FDR and normalized $\beta_{coefficient}$ for the result of our differential gene expression analysis for every treatment and timepoint relative to vehicle control using a formula of "*expression ~ treatment + replicate*". The results of all test were combined and subjected to multiple hypothesis testing using the Benjamini-Hochberg procedure. **f)** UpSetR plot of the overlap of differentially expressed genes across each of our tests.

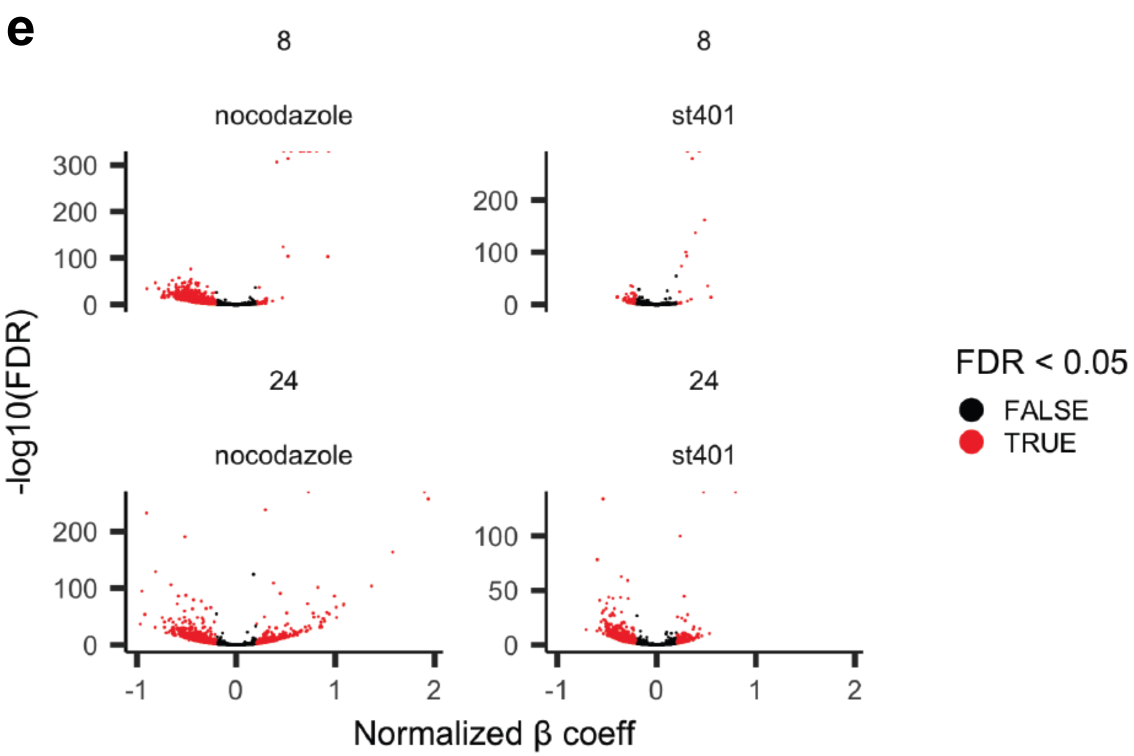

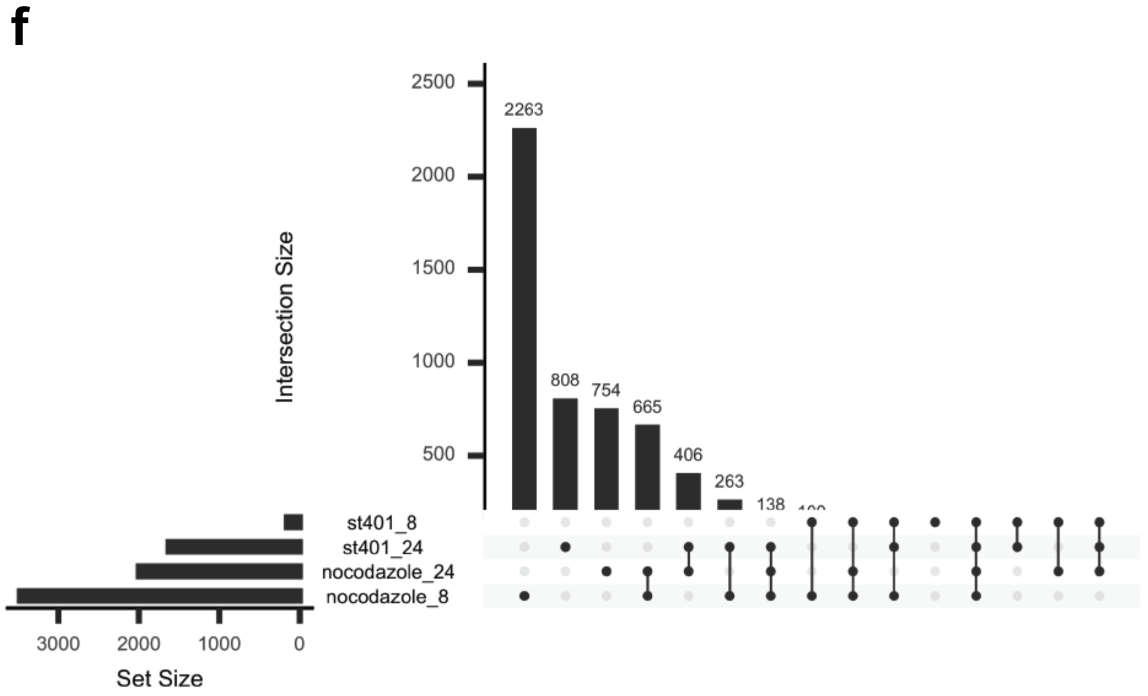

Supplementary figures S5g-h: Gene-enrichment analysis at 24h using Metascape. After scRNAseq analysis, genes with statistical significance differences were divided into 4 groups: genes upregulated (up) in ST and downregulated (down) in NOC **(gi)**, genes up in ST and up in NOC **(gii)**, genes down in ST and down in NOC **(hi)** and genes down in ST and up in NOC **(hii)**. These groups were then analyzed using the platform Metascape for gene enrichment analysis.

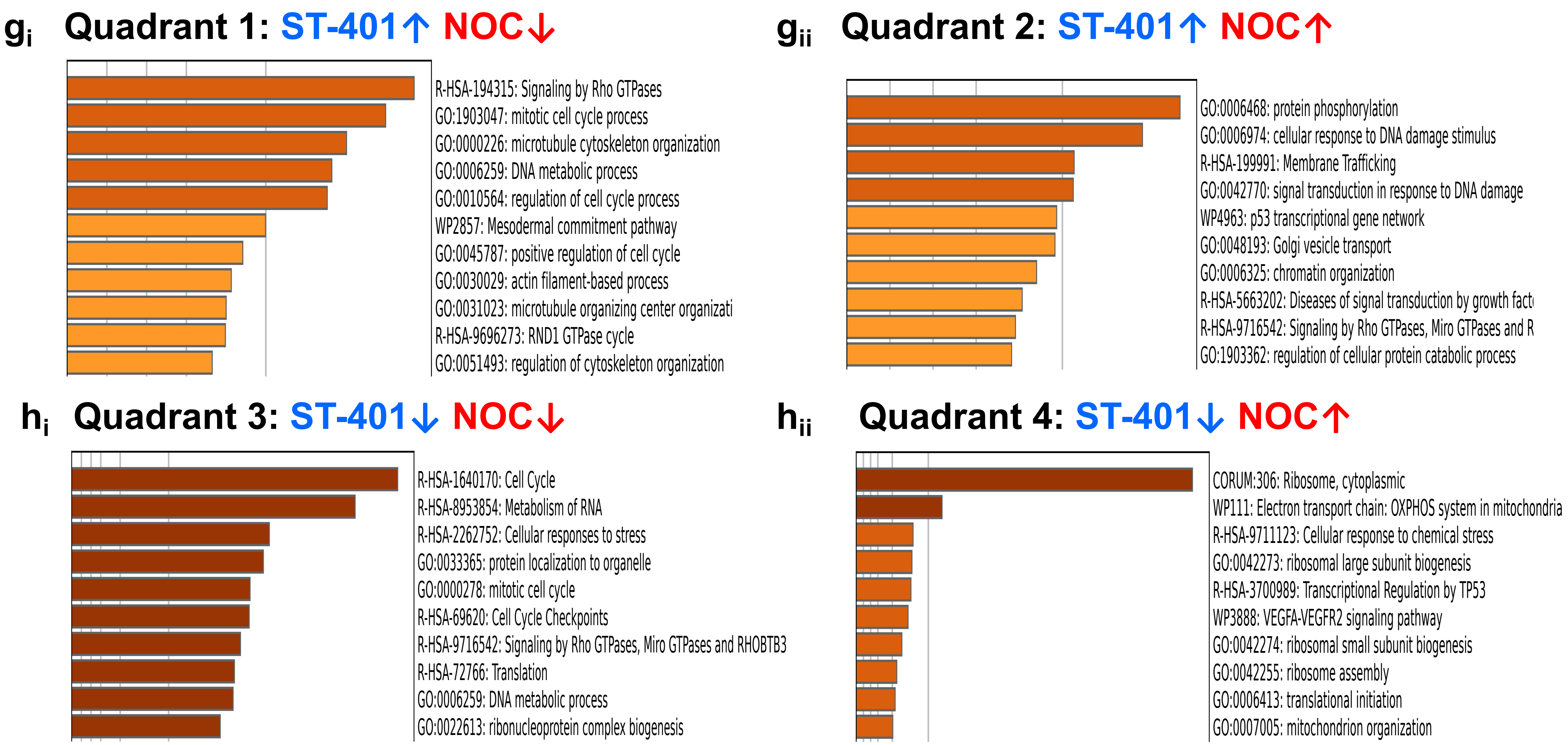

Supplementary figure S5i: Dimensionality reduction using Principal Component Analysis (PCA) analysis of the cluster distribution of CTR, NOC and ST-401 treated HCT116 cells for 8h and 24h. Clusters confirmed more complex cluster distributions between CTR, NOC and ST-401 treated for 24h compared to 8h.

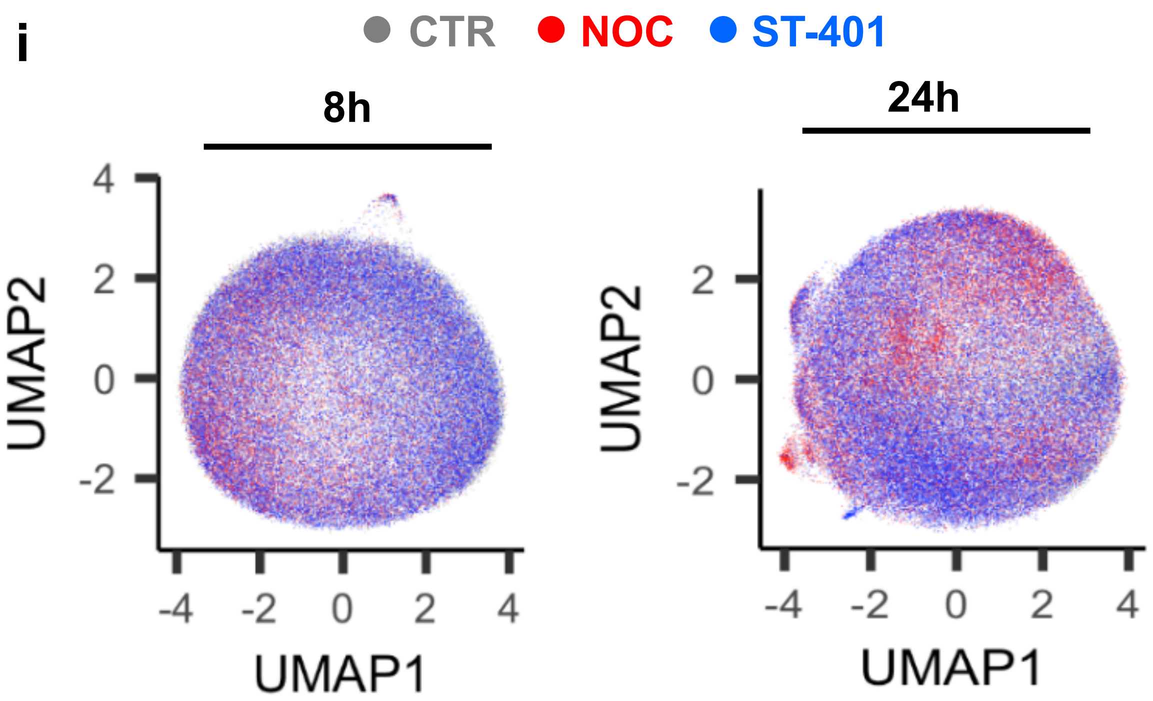

Supplementary figure S6a-b: Ponceau Red staining of gels shown in Figure 6. **a)** Representative ponceau Red stain of gel used for Puromycin analysis. **b)** Representative ponceau Red stain of gel used for Puromycin analysis.

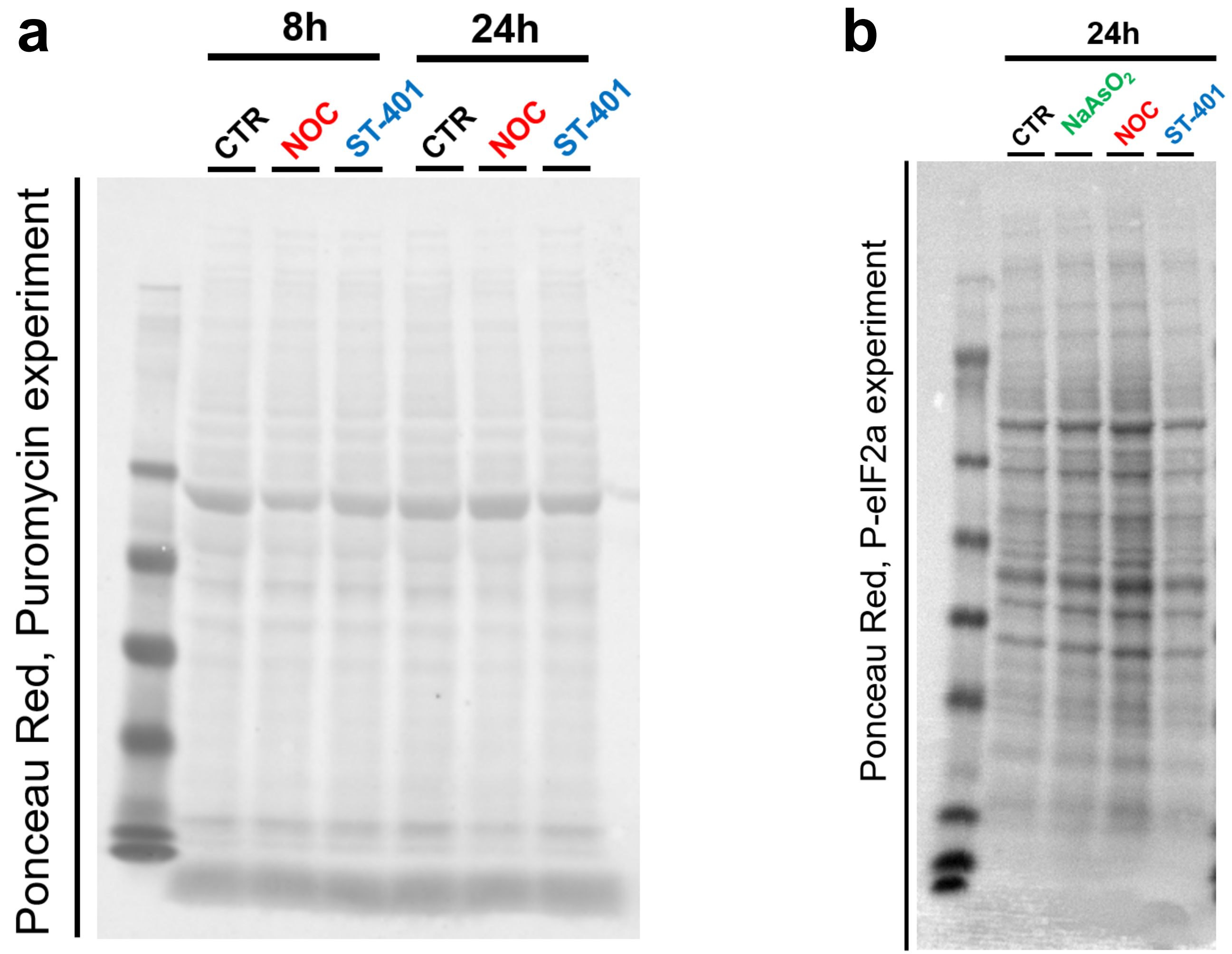

Figure S7a-b: NOC (100 nM) and ST-401 (100 nM) treatment for 24h affects spare capacity and proton leak in HCT-116 cells. **a-b)** Spare capacity and proton leak were calculated from Figure 7e. Statistics: Data are shown as mean ± s.e.m. of 5 independent experiments. Ordinary one-way ANOVA analysis resulted in ***=p<0.001 compared to CTR.

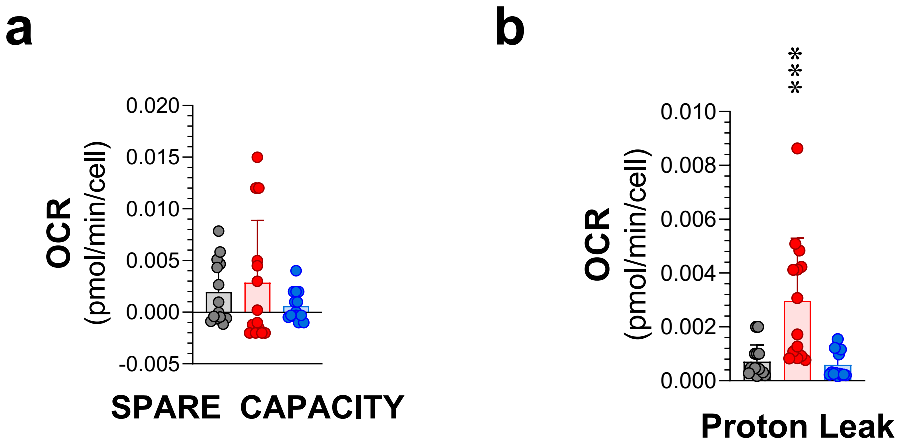

Figure S8a-b: NOC (100 nM) and ST-401 (100 nM) treatment for 24h affects non-mitochondrial respiration and maximal respiration as measured by Cell Mito Stress Test, in SF-539 and SNB-19 cells. Analysis from results shown in Figure 8e&g.

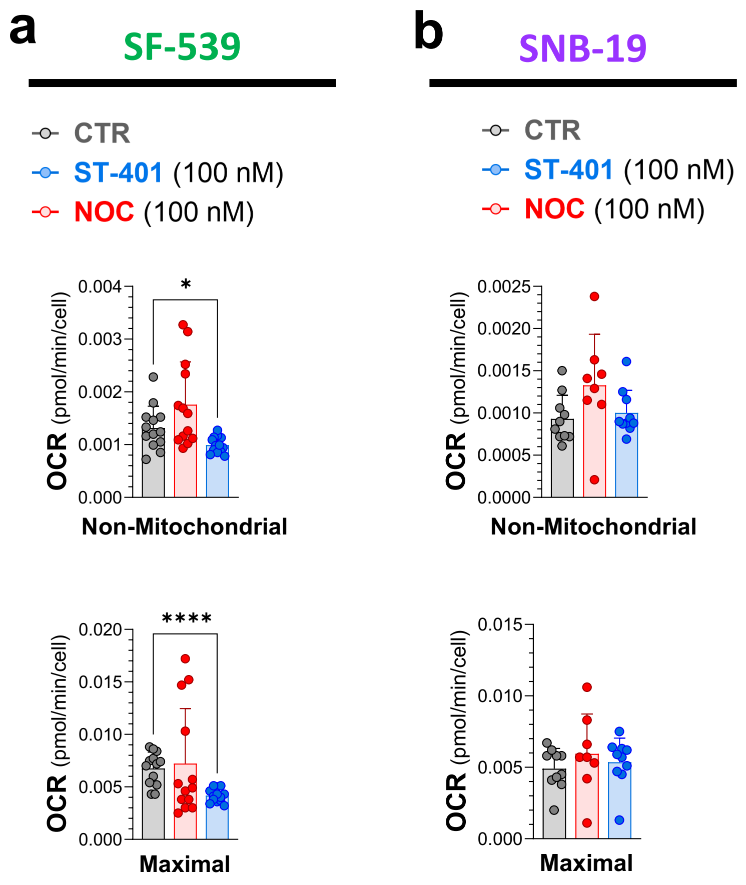

Figure S9a-b: NOC (100 nM) and ST-401 (100 nM) treatment for 24h affects mRNA encoding for immune responses in HCT-116 cells. **a-b)** scRNAseq data showing the expression of select mRNAs involved in cancer immunotherapy, as well as HLA genes. Analysis for data presented in Figure 5f.

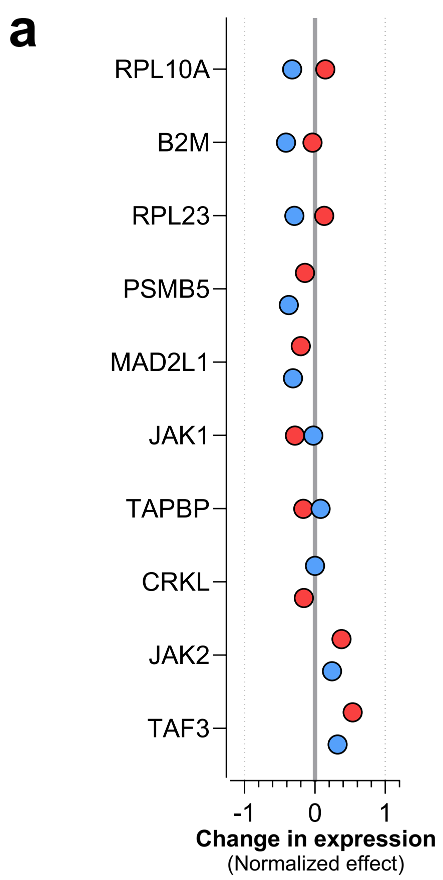

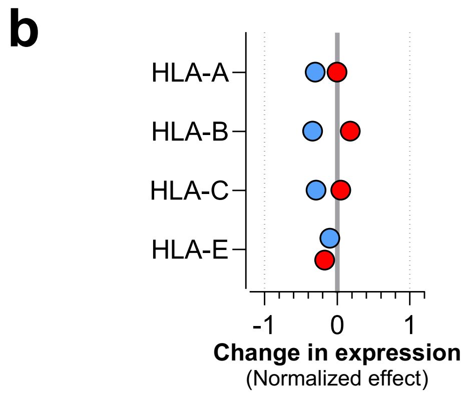

Graphical Abstract

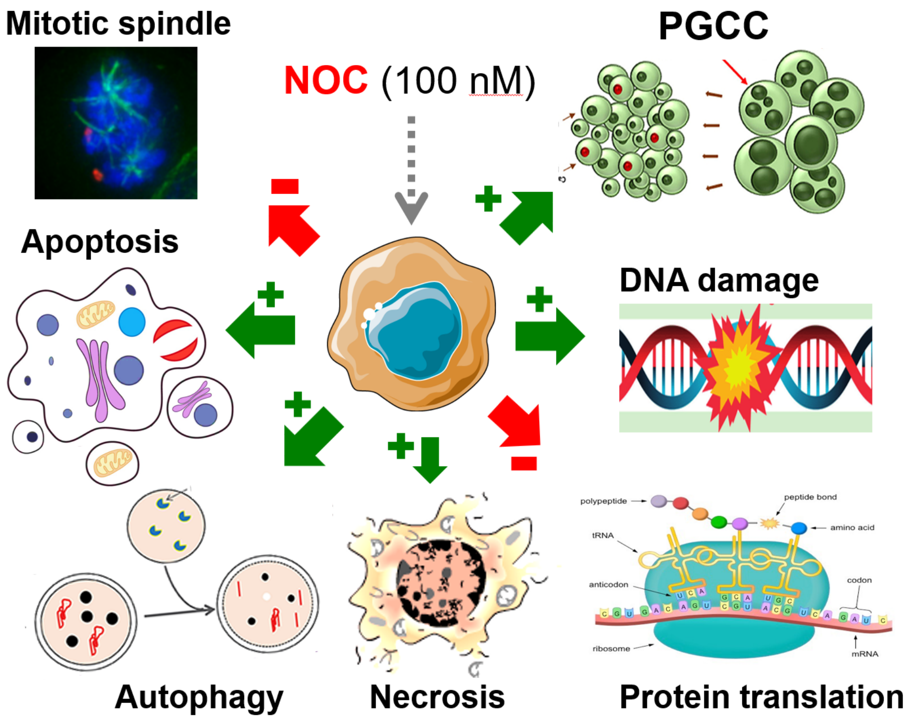

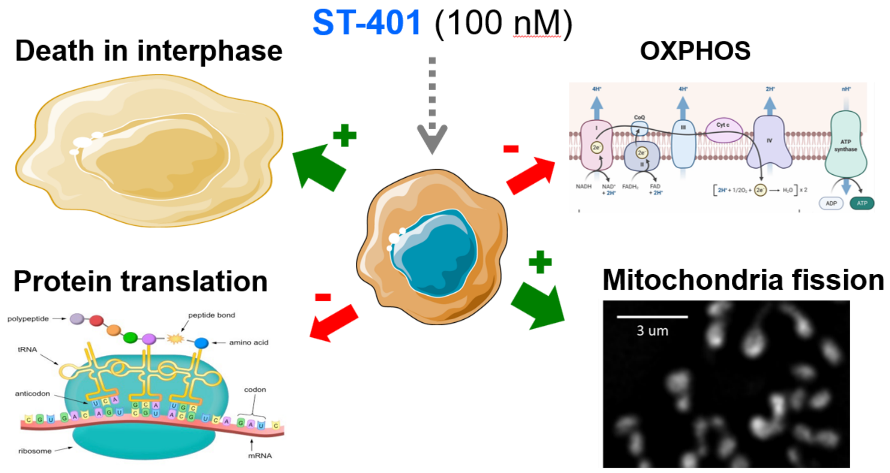

Diagram depicting that NOC treatment of HCT116 cells leads to death in mitosis, increases autophagy, necrosis and apoptosis, reduces protein translation and triggers the generation of PGCCs.

Diagram depicting that ST-401 treatment of HCT116 cells leads to death in interphase, transient reduction in protein synthesis, mitochondria fission and reduction in OXPHOS.
